# Early Detection and Monitoring of Anastomotic Leaks via Naked Eye‐Readable, Non‐Electronic Macromolecular Network Sensors

**DOI:** 10.1002/advs.202400673

**Published:** 2024-05-22

**Authors:** Alexander Jessernig, Alexandre H.C. Anthis, Emilie Vonna, Jachym Rosendorf, Vaclav Liska, Jeannette Widmer, Andrea A. Schlegel, Inge K. Herrmann

**Affiliations:** ^1^ Nanoparticle Systems Engineering Laboratory Institute of Energy and Process Engineering (IEPE) Department of Mechanical and Process Engineering (D‐MAVT) ETH Zurich Sonneggstrasse 3 Zurich 8092 Switzerland; ^2^ Particles‐Biology Interactions Laboratory Department of Materials Meet Life Swiss Federal Laboratories for Materials Science and Technology (Empa) Lerchenfeldstrasse 5 St. Gallen 9014 Switzerland; ^3^ Department of Surgery Faculty of Medicine in Pilsen Charles University Alej Svobody 923/80 Pilsen 32300 Czech Republic; ^4^ Biomedical Center Faculty of Medicine in Pilsen Charles University Alej Svobody 1655/76 Pilsen 32300 Czech Republic; ^5^ Department of Surgery and Transplantation Swiss HPB Centre University Hospital Zurich Zürich 8091 Switzerland; ^6^ Transplantation Center Digestive Disease and Surgery Institute and Department of Immunity and Inflammation Lerner Research Institute Cleveland Clinic 9620 Carnegie Ave Cleveland OH 44106 USA; ^7^ The Ingenuity Lab University Hospital Balgrist Balgrist Campus Forchstrasse 340 Zurich 8008 Switzerland; ^8^ Faculty of Medicine University of Zurich Rämistrasse 74 Zürich 8006 Switzerland

**Keywords:** device, drain fluid, postoperative complications, sensing, wearables

## Abstract

Anastomotic leakage (AL) is the leaking of non‐sterile gastrointestinal contents into a patient's abdominal cavity. AL is one of the most dreaded complications following gastrointestinal surgery, with mortality rates reaching up to 27%. The current diagnostic methods for anastomotic leaks are limited in sensitivity and specificity. Since the timing of detection directly impacts patient outcomes, developing new, fast, and simple methods for early leak detection is crucial. Here, a naked eye‐readable, electronic‐free macromolecular network drain fluid sensor is introduced for continuous monitoring and early detection of AL at the patient's bedside. The sensor array comprises three different macromolecular network sensing elements, each tailored for selectivity toward the three major digestive enzymes found in the drainage fluid during a developing AL. Upon digestion of the macromolecular network structure by the respective digestive enzymes, the sensor produces an optical shift discernible to the naked eye. The diagnostic efficacy and clinical applicability of these sensors are demonstrated using clinical samples from 32 patients, yielding a Receiver Operating Characteristic Area Under the Curve (ROC AUC) of 1.0. This work has the potential to significantly contribute to improved patient outcomes through continuous monitoring and early, low‐cost, and reliable AL detection.

## Graphical abstract

Anastomotic leakage (AL) is the leaking of pathogenic, non‐sterile gastrointestinal contents into the abdominal cavity of a patient, with mortality rates of up to 27%. In this work, a naked eye‐readable, electronic‐free sensor for the continuous monitoring of drain fluid enzymes and early detection of AL is introduced.

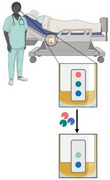

## 1 Introduction

**Figure 1:**
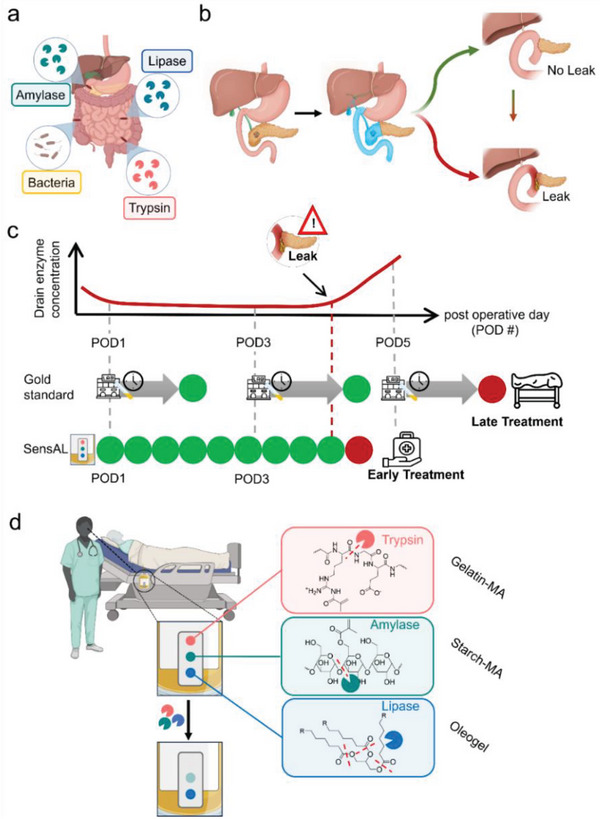
Schematic of a novel biosensor that enables rapid and facile naked eye detection of AL. a) Enzymes present in the GI tract. b) Schematic of a pancreatic surgery including anastomotic reconnections prone to leaking. In case of leaking anastomotic reconnections, non‐sterile, digestive enzyme‐containing contents of the GI tract enter the abdominal cavity causing complications and infections. c) Comparison of current gold standard drain fluid analysis (amylase quantification at discrete time points, typically on postoperative days (POD) 1, 3, and 5) with the new continuous monitoring approach based on macromolecular network sensors (termed SensAL). By using a continuous monitoring approach, leaks can be detected faster and treatment initialized earlier. d) Schematic illustration of SensAL deployment in a hospital setting. The sensor can be mounted in pre‐existing drainage bags that are routinely placed at the patient's bedside. The sensor consists of structurally colored sensing elements that are responsive to all major digestive enzymes found inside the human GI tract. In the absence of enzymes the sensors remain unchanged, while in the presence of enzyme, the macromolecular network sensors change color indicating the potential presence of an AL. Figure created using Biorender.com.

Anastomotic reconstruction of the organs in the gastrointestinal (GI) tract is widely employed by surgeons on a daily basis during many routine interventions, including gastric‐bypass, resections for inflammatory bowel disease and cancer resection surgeries.^[^ ^1^ ^]^ Approximately 14 million gastrointestinal surgeries are performed worldwide each year.^[^ ^2^, ^3^ ^]^ These interventions, while often live‐saving, simultaneously expose patients to the potential risk of an anastomotic leak (AL). The incidence of AL fluctuates based on factors such as the anastomotic site, the specific surgical procedure (esophageal anastomosis (leak rate ≈20%),^[^ ^4^ ^]^ pancreaticojejunal anastomosis (leak rate ≈10%),^[^ ^5^ ^]^ coloanal anastomoses (leak rate up to 19%),^[^ ^6^ ^]^ and the patient's personal predisposition, such as patient weight, cardiovascular diseases, patient lifestyle (alcohol, smoking etc.) as well as preoperative chemotherapy.^[^ ^7^ ^]^ The consequences of a leak are often severe and can be fatal,^[^ ^8^, ^9^ ^]^ due to the leaking of digestive, non‐sterile fluids into the abdominal cavity of a patient (**Figure** **1**a,b). Swift detection and intervention are crucial to prevent life‐threatening complications and enhance the patient's recovery. Mortality rates, ranging from 13% to 27%, predominantly result from the progression to sepsis and septic shock, primarily due to delayed detection. This delay arises from the absence of specific and early symptoms associated with AL and the lack of technologies that enable continuous monitoring and could facilitate early detection and diagnosis.^[^ ^10^, ^11^, ^12^ ^]^ Additionally, the occurrence of late‐onset leaks is non‐negligible,^[^ ^13^, ^14^ ^]^ further complicating adequate patient monitoring efforts. To this day there is no rapid and facile detection method available for the early identification of impending complications, including AL, which is why the average time period to detect anastomotic insufficiency is between 5 and 8 days.^[^ ^15^ ^]^

In current hospital practice, drains are routinely placed for the postoperative management of patients at risk for AL.^[^ ^16^ ^]^ These drains draw fluid from the surgical site and divert it to the outside of the patient's body. However, drain fluid analysis is still underdeveloped and patient condition is oftentimes assessed based on drain fluid coloration (red indicating bleeding, yellow/green, and/or cloudiness suggesting infection and/or the presence of bile in the drain). Additionally, patient blood is routinely analyzed for inflammatory biomarkers, including C‐reactive protein (CRP) as well as leukocyte count and results are compared to the baseline of the patient prior to surgery and the reference range of healthy individuals. Abnormal clinical and laboratory results in conjunction with patient discomfort are frequently accompanied by cross sectional imaging such as computer tomography (CT), of the abdomen. However, CT‐based assessment lacks the ability to detect smaller leaks and exposes the patient to harmful ionizing radiation. The aforementioned methods while providing decent negative predictive values,^[^ ^17^, ^18^, ^19^ ^]^ are non‐specific, require active human intervention, are labor‐ and resource‐intensive, and generally do not have the power to reliably predict an AL.^[^ ^20^, ^21^ ^]^

Given the limited effectiveness of existing detection methods, researchers have explored alternative experimental strategies for AL detection. One such approach is the placement of bio‐degradable electrodes around the suture site to measure changes in impedance after contact with gastric fluid caused by an AL.^[^ ^22^ ^]^ However, this approach is invasive and limited to the detection of gastric fluid leaks occurring near the electrode. More recently, the presence of amylase, an enzyme responsible for carbohydrate digestion in humans, in surgical drain fluid has been identified as a promising biomarker with a good predictive value for anastomotic leaks. Studies where drain amylase levels were detected through an amylase assay in a centralized lab have shown drain amylase to be a good indicator of pancreatic or small bowel AL on postoperative days 3 to 7.^[^ ^23^ ^]^ Drain amylase also proves effective independently of the surgical site (ranging from esophagus to rectum), or the patient's underlying health conditions and comorbidities.^[^ ^24^ ^]^ Nonetheless, a drawback inherent to this approach lies in the prerequisite for a hospital to possess the appropriate laboratory infrastructure, resources, and capacity to determine drain amylase levels in a close‐meshed manner. This requirement can be particularly challenging for smaller hospitals and in less developed parts of the world. Additionally, the drain fluid sample needs to be manually collected and sent to a laboratory, and therefore enables only discontinuous monitoring at predefined time points (Figure 1c). A recently published study used an amylase‐specific antigen assay for spectroscopic leak detection.^[^ ^25^ ^]^ Although the findings were promising, this technique necessitates specialized equipment, involving an antibody‐coated sensor chip, a light source, and an optical spectrophotometer. Additionally, the drain fluid needs to be diluted, and hence does not adequately address the clinical needs. Particularly in strenuous times when hospitals are understaffed, this discontinuous monitoring, which requires active involvement of hospital staff, can significantly increase the risk of late AL detection, negatively impacting patient recovery and outcomes. While amylase has become an established biomarker,^[^ ^26^ ^]^ the diagnostic potential (as a function of type of surgery) for trypsin and lipase are yet to be comprehensively analyzed in clinical settings.

Motivated by the limitations exhibited by the state‐of‐the‐art AL detection techniques, we conceived a straightforward approach for optically identifying AL leaks in real‐time, devoid of any necessity for specialized equipment (termed SensAL, Figure 1d). Through first‐of‐its‐kind bioinspired macromolecular network sensing elements that were designed to be selectively responsive to all major digestive enzymes found in the GI tract (trypsin, amylase, lipase) and placing them in surgical drains, we demonstrate reliable detection of leaks with high precision and sensitivity in a continuous manner directly at the patient's bedside (Figure 1c,d). Digestive enzymes offer enhanced detection capabilities compared to non‐enzymatic biomarkers due to their catalytic nature. When an enzyme biomarker is present, even in small quantities, it can catalyze the conversion of large amounts of substrate into product. This amplification effect enables the detection of potential AL at earlier stages than might be possible with non‐enzymatic biomarkers, and directly at the bedside. Additionally, by introducing structural colors or pigments as a coloring agents, the presence of enzymes in drain fluid (indicative of a developing AL) can be detected through non‐electronic naked eye detection. This sensor design marks a decisive advancement in enabling early, electronic free AL detection and paving the way to close‐meshed monitoring, as it eliminates the laborious, costly and time‐consuming limitations posed by the current gold standard. Additionally, the simple naked eye readout opens a new avenue by giving doctors the ability to detect the presence of enzymes at the patient bedside, thereby increasing patient safety and streamlining hospital procedures as well as home‐monitoring, thus safeguarding time and resources.

## 2 Results and Discussion

### 2.1 Macromolecular Network Design and Performance

**Figure 2:**
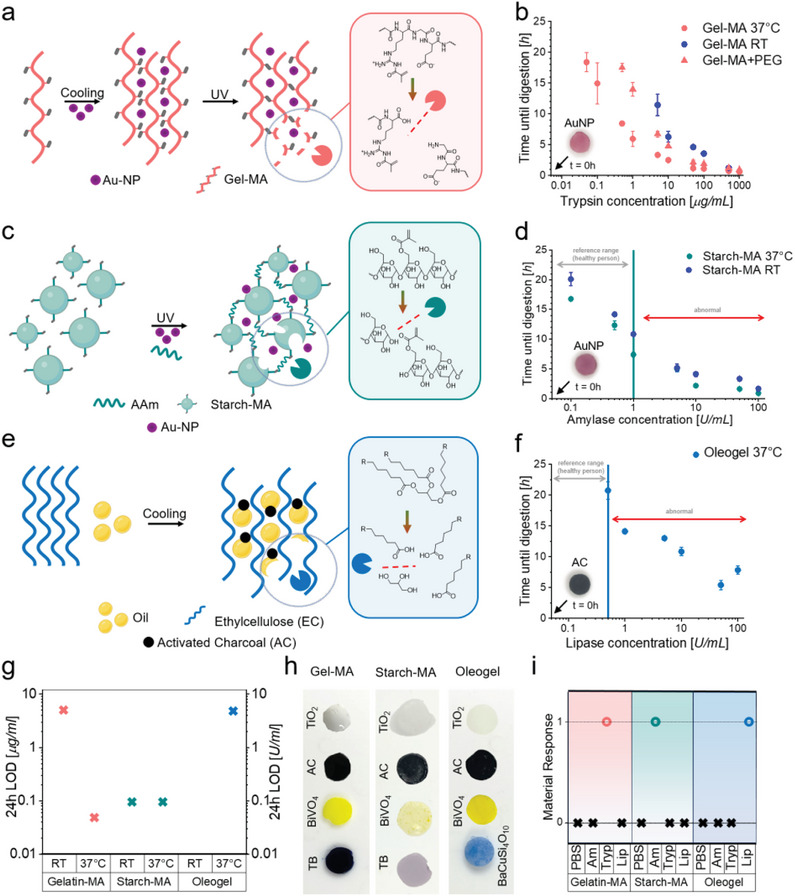
Synthesis and kinetics of enzyme responsive macromolecular network sensor materials. a) Design and preparation of structurally colored trypsin responsive Gel‐MA sensing elements. b) Kinetics of Gel‐MA macromolecular network sensors at different temperatures and with additional crosslinking. c) Design and preparation of structurally colored amylase responsive Starch‐MA sensing elements. d) Kinetics of Starch‐MA sensing elements at different temperatures. e) Design and preparation of lipase responsive oleogel sensors. f) Kinetics of oleogel sensing elements at body temperature. g) Lower limit of detection (LOD) of the sensing elements at room temperature (RT) and body temperature (37 °C). h) Left: Gel‐MA sensing elements colored with titania, charcoal, BiVO_4_ and trypan blue to provide different sensing element coloration. Center: Starch‐MA sensing elements colored with titania, charcoal, BiVO_4_ and trypan blue to provide different sensing element coloration. Right: Oleogel sensing elements colored with titania, charcoal, BiVO_4_ and BaCuSi_4_O_10_ to provide different sensing element coloration. i) Response of the sensing elements to different enzymes. All macromolecular network sensors show high selectivity, as they only respond to their corresponding enzymes. Value 0 indicates no reaction (no response), value 1 indicates reaction (response) in presence of the indicated enzyme. PBS: phosphate buffered saline, Am: amylase, Tryp: trypsin, Lip: lipase. Data shown as mean ± standard deviation. All experiments were repeated at least 3 times using independent samples (N = 3).

**Figure 3:**
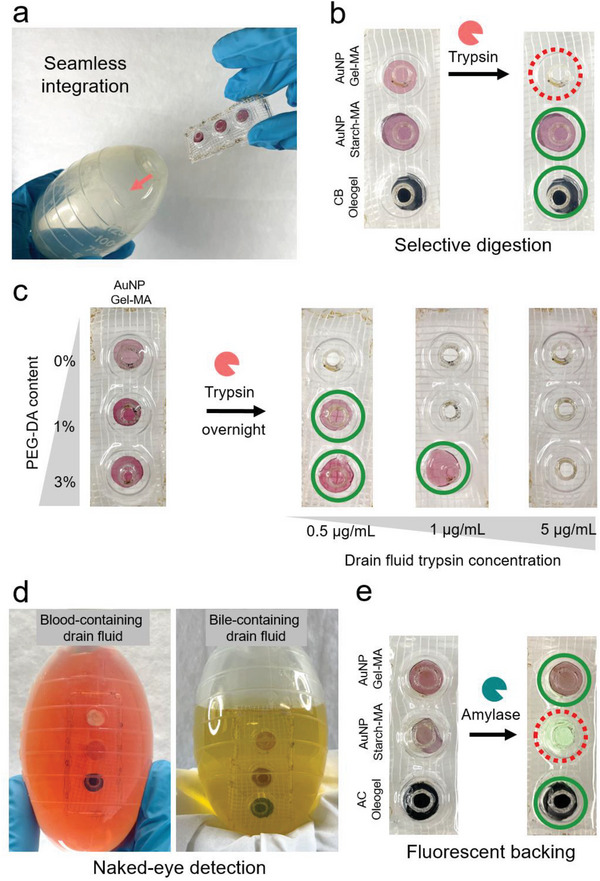
Prototype design, device intrgration and additional features. a) Compatibility of the sensor array with commonly employed drainage bags. Through modifications to the backing the sensor can be used with any type of drainage bag. b) Naked eye readout of trypsin presence in drain contents. The disappearance of the structurally colored Gel‐MA sensing element indicates the presence of trypsin. Non trypsin responsive sensing elements remain intact, indicating high sensor selectivity. c) Semi quantitative sensor readout. Adding different amounts of PEG‐DA influences digestibility and allows a semi‐quantitative readout of enzyme concentration in drain fluids. d) Enzyme sensor in a drain bag filled with simulated leak fluid. Sensors are still well visible allowing naked eye detection in clinically relevant, colored fluids. e) Sensor with modified backing. Adding different colors to the backing or even using a fluorescing backing can further facilitate naked eye discernibility in the case of strongly colored drain fluids (containing blood or bile). All experiments were repeated at least 3 times using independent samples (N = 3).

Since digestive enzymes are only found inside the GI tract in healthy individuals, detecting them in the drainage fluid is indicative of an AL.^[^ ^24^ ^]^ We therefore designed enzyme‐responsive macromolecular network sensing elements for the sensitive and specific detection of digestive enzymes in abdominal drain fluid. Overall, three different macromolecular network materials were developed, each of them responsive to a different digestive enzyme (trypsin, amylase and lipase). First, we designed and synthesized a trypsin responsive macromolecular network based on gelatin (**Figure** **2**a). In fact, trypsin concentration inside the GI tract can be relatively high (>500 µg mL^−1^), especially after protein‐rich food consumption.^[^ ^27^ ^]^ Its presence outside of the GI tract and in the peritoneal fluid, however, should be a definitive indicator of leakage, as trypsin is a major excretion of the GI tract.^[^ ^28^ ^]^ Monitoring trypsin activity can thus be an effective method for early anastomotic leak detection. As a natural substrate of trypsin and a major constituent of mammalian skin, gelatin has high biocompatibility, forms gels easily, and most importantly, is readily degraded by trypsin. To improve the stability and tailor the macromolecular network properties, amine and guanidine residues present in the gelatin amino acid backbone were methacrylated using methacrylic anhydride, resulting in the formation of gelatin methacrylate (Gel‐MA) (Figure S1, Supporting Information). The degree of MA substitution can be controlled by the amount of methacrylic anhydride added and plays an important role in the crosslinking density of the final macromolecular network (gel).^[^ ^29^, ^30^, ^31^ ^]^ To achieve naked eye detection, the macromolecular network gels were structurally colored, for example, by adding Gel‐MA to a gold nanoparticle suspension at elevated temperatures. Following the addition of the photoinitiator Lithium phenyl‐2,4,6‐trimethylbenzoylphosphinate (LAP), the warm Gel‐MA solution was poured onto a glass surface to form a thin film of a few millimeters and cooled to 4 °C. By cooling the gelatin, a physically crosslinked hydrogel network is formed. However, as gelatin readily re‐dissolves at temperatures ≈30 °C (e.g., the temperature of the drain fluid after it leaves the body), a second chemical crosslinking step was introduced to exclude the possibility of temperature‐induced false positives. The photo‐induced polymerization of the methacrylate groups using LAP creates a double network hydrogel that provides the desired stability with the color giving elements (e.g., gold) entrapped within the network (Figure 2a). Gold nanoparticles were selected as they display structural coloration through surface plasmon resonance (SPR), which involves the collective oscillation of free electrons on the nanoparticle surface induced by light. SPR is highly sensitive to changes in the local environment, including the refractive index of the surrounding medium, the presence of nearby (bio)molecules, temperature, pH, as well as distance between gold nanoparticles.^[^ ^32^ ^]^ By changing the interparticle distance as is the case after digestion, the color disappears within the sensor element while the coloration of the surrounding drain fluid remains unaffected (due to dilution and the resulting large distance between gold nanoparticles). Therefore, the sensor in its current form delivers a readout through the disappearance of the sensing element and vanishing of intense coloration. The Gel‐MA‐based protease‐responsive macromolecular network exhibits rapid response times as can be seen in Figure 2b where the kinetics of the protease responsive sensing element are shown. The lowest trypsin quantity detected at room temperature (RT) was 5 µg mL^−1^ (lower limit of detection LLOD_RT_), while the lowest detectable concentration at body‐temperature (BT) was LLOD_BT_ = 0.05 µg mL^−1^ (Figure 2g). The dependence of the LLOD on the temperature is not unexpected due to the fact that the activity of enzymes is oftentimes highly temperature dependent and human digestive enzymes evolved to function optimally at body‐temperature.^[^ ^33^, ^34^ ^]^ Interestingly, by adding an additional crosslinker (here poly(ethylene glycol) diacrylate (PEG‐DA, 700 Da)), the digestibility of the macromolecular network can be further tuned in order to make the macromolecular network more robust toward enzymatic digestion, and hence tailor the sensitivity of the response to the diagnostically relevant range (clinically significant threshold of 1 U mL^−1^ of amylase and 0.5 U mL^−1^ of lipase,^[^ ^35^, ^36^, ^37^ ^]^ see Figures 2b and **3**c). Since drain fluid sample analysis is still in its early days of development, there is little literature on the diagnostic utility of trypsin detection in AL patients. However, a recent study suggest that elevated trypsin and chymotrypsin levels can be predictive of postoperative pancreatic fistula after pancreas resections.^[^ ^38^ ^]^ Based on the above, its potential clinical utility seems promising and substantiated (vide infra).

With drain amylase emerging as the gold standard biomarker for AL detection,^[^ ^24^ ^]^ we developed a macromolecular network sensing element capable of detecting the presence of amylase in drain fluids quickly and with high sensitivity. We designed a macromolecular network sensor crosslinked by a natural substrate of amylase: starch. Starch is a long chain carbohydrate that is found in most plants and an essential component of human nutrition. It generally comes in water‐insoluble granules that swell up when heated.^[^ ^39^ ^]^ There are several examples for starch‐based hydrogels, however, only few of them rely on chemical crosslinking.^[^ ^40^ ^]^ Since chemical and mechanical stability of the macromolecular network sensing element in the absence of enzymes is key for the envisioned application, we chemically modified starch to contain crosslink‐able methacrylate groups using a base catalyzed procedure (Figure S2, Supporting Information).^[^ ^41^ ^]^ Having slightly elevated temperatures during methacrylation ensures better functionalization of alcohol groups inside the granules and therefore a higher degree of desired functional groups.^[^ ^42^ ^]^ Starch granules are generally insoluble in water, however at elevated temperatures granules swell due to increased water uptake, which leads to increased reagent influx and therefore results in higher degree of functionalization^[^ ^43^ ^]^ (Figure 2c). By heating the gel mix up to 80 °C, we ensured granule swelling (visible by a drastic increase in viscosity) through gold nanoparticle solution intake. UV‐induced polymerization then led to a gel where semi‐swollen granules are connected by acrylamide chains giving an amylase responsive hydrogel that displays a gold nanoparticle induced structural color. The kinetics and the sensitivity of the amylase‐responsive sensing element indicated high sensitivity and only minimal temperature dependence. The LLOD_RT_ and LLOD_BT_ for amylase using the starch‐MA macromolecular network sensor were 0.01 U mL^−1^ (Figure 2g). The sensor was able to detect a commonly referred clinically significant threshold of 1 U mL^−1^ of amylase^[^ ^35^, ^36^ ^]^ within a few hours or less at room temperature.^[^ ^44^ ^]^ These properties establish the prerequisites for detecting potential AL, for example, overnight, without the need for expensive equipment or specialized personnel.

To fully monitor all major digestive enzymes found in the human GI‐tract, we also designed and developed a lipase responsive sensing element (Figure 2e). Lipid digestion is a complex interface‐dependent process that involves substrates in droplet form and several different lipases and cofactors (e.g., co‐lipase, bile salts).^[^ ^45^, ^46^ ^]^ Therefore it is paramount to keep the responsive sensing element as close to the natural substrates of lipases as possible. Most dietary fats have melting points around room temperature, however, for a suitable sensor design, it is necessary to preserve the solid structure and mechanical integrity even at elevated temperatures. To achieve this, we adapted a concept from food science, where oil is packaged into a physical gel (oleogel).^[^ ^47^, ^48^ ^]^ Oleogels are typically employed for the solidification of lipids and as replacement of trans and saturated fats. Figure 2e shows a schematic of the approach used for our lipase responsive sensing element. Plant oil (coconut fat) was mixed with ethylcellulose (EC) and a colorant (e.g., active charcoal (black), titania (white), BiVO_4_ (yellow), BaCuSi_4_O_10_ (blue), see also Figure 2h) at elevated temperatures, which causes the EC to dissolve in the hot fat. The homogenous mixture was then poured onto a glass plate causing a rapid drop in temperature. During the cooling process the EC formed a quasi‐crystalline physical network encapsulating the fat droplets. This encapsulation results in favorable heat stability of oleogels,^[^ ^49^ ^]^ and enables the creation of oleogel‐based sensing elements. Using these oleogel sensing elements we are able to detect lipase concentrations as low as LLOD_BT_ = 0.5 U mL^−1^ at 37 °C (Figure 2g). Such a threshold value coincides with the clinically interesting enzyme concentration that has been considered to be a good indicator of AL after pancreatic resection.^[^ ^37^ ^]^ The response time of the lipase sensor increases at RT, and sensing elements needed >24 h to fully react with the total number of enzymes present in the drain fluid volumes used for these measurements. Lipase activity is also highly bile salt dependent,^[^ ^50^, ^51^ ^]^ and thus the lipase‐responsive macromolecular sensors were the least robust among the three. However, since the drain bags are often attached to patients for multiple days, a lipase responsive sensing element can serve as a long‐term monitoring sensor and can be indicative of large leaks.

Notably, all the developed macromolecular network sensing elements showed high specificity for the respective enzymes (Figure 2i). Macromolecular network sensing elements did not cross‐react with non‐corresponding enzymes, even after extended durations of contact (>72 h). The response time of the sensor elements is dictated by their composition and especially the network structure (vide infra) as well as the enzyme concentration in the drain fluid. Regarding sensitivity and operation within clinically relevant ranges, both the trypsin and amylase‐responsive sensors demonstrated particularly promising sensitivities and rapid response times of a few hours or less at diagnostically relevant enzyme concentrations. With regard to sensor robustness, pH and temperature‐dependence were assessed covering clinically relevant pH and temperature ranges. Previous studies showed that the pH value of the drain fluids only shows minimal fluctuations between pH 7 and 8.^[^ ^52^ ^]^ The amylase, lipase and trypsin activity within this range is not affected by pH (Figure S10, Supporting Information). With regard to temperature‐dependence, the trypsin and amylase sensors show a modest temperature dependence and slower reaction at room temperature compared to body temperature (e.g., 1.5 h instead of 1 h for levels of 100 U mL^−1^ amylase, and 3.5 h vs 1 h for 100 µg mL^−1^ trypsin), while the lipase sensor only shows clinically feasible reaction times at body temperature. While reaction times may vary as a function of temperature, there remains to be a projected benefit in early detection compared to the gold standard of discontinuous monitoring every second day (enzyme detection within few hours vs days).

### 2.2 Electronic‐Free Macromolecular Network Sensors – Design and Assembly

Before the macromolecular network sensing elements could undergo clinically relevant diagnostic performance evaluation, they had to be integrated into a sensor array suitable for performance assessment. Our goal was to develop a sensor prototype meeting a range of essential criteria to ensure its clinical applicability. First, to achieve optimal reactivity, the contact area between the sensing elements and the drain fluid must be maximized. Second, the sensor needs to be integratable and easily adaptable to the diverse range of drainage bags routinely used in standard hospital care. We accomplished this by designing an array that arranges the various enzyme‐responsive sensing elements on a supporting surface, securing their placement (Figure 3a). To optimize liquid interaction with the sensing elements while minimizing diffusion constraints, we strategically designed the backing. This backing features perforations on the front surface and integrates a permeable net on the reverse side, maximizing the sensing elements' contact area with the liquid and ensuring effective sensing capabilities and maximal sensitivity. This configuration also facilitates the sensor array's seamless insertion into various drainage bags, accommodating potential variations in clinical setups (Figure 3a). The high selectivity of the sensor array is demonstrated in Figure 3b. The left image displays the macromolecular network sensor before integration into the drain bag, while the right image shows the sensor after incubation in trypsin‐containing physiological fluid. Only the trypsin‐responsive sensing element undergoes digestion, while in contrast, the amylase and lipase‐responsive sensing elements remain unchanged. Crucially, the selection of sensing elements for the array is highly flexible. Aside from the integration of a trypsin‐responsive, an amylase‐responsive, and a lipase‐responsive sensor, the array can also accommodate sensing elements with varying levels of sensitivity. While the presence of enzymes in the drain fluid is indicative of a leak, the severity of an AL often correlates with enzyme concentration of the effluent, and hence the sensor configuration can also enable semi‐quantitative readouts indicative of leak severity. Additionally, remnant enzymes may be present in the first days after surgery due to insufficient flushing of the abdominal cavity after surgery. To account for such events, the sensitivity (i.e., reactivity) of the macromolecular sensing elements can be tailored to the clinical needs. For example, by introducing varying quantities of poly(ethylene glycol)diacrylate (PEG‐DA (700 Da)) to the trypsin‐responsive macromolecular networks (Figure 3c), their susceptibility to digestion can be made trypsin concentration dependent. With increasing PEG‐DA amounts, the macromolecular network sensing elements become less susceptible to digestion. Leveraging this approach, opens the door to a semi‐quantitative assessment of enzyme concentrations (Figure 3c). In this case, the sensor was loaded with three different Gel‐MA macromolecular network sensing elements, containing 0%, 1%, and 3% PEG‐DA colored with gold nanoparticles. The simulated leak fluid in the drain was spiked with different concentrations of trypsin and the system was kept at RT overnight. The sensing elements devoid of PEG‐DA underwent digestion at low enzyme concentrations (0.5 µg mL^−1^). In contrast, the sensing elements incorporating 3% PEG‐DA necessitated a tenfold higher enzyme concentration to undergo complete digestion within the same period of time.

Furthermore, we aimed to develop a sensor with a reliable naked eye readout. This can be either achieved by the use of structural colorants or alternatively, via the use of pigments. Using structural coloring, for example, based on gold nanoparticles, a bright color can be given to the macromolecular network sensing elements making them readily discernible from the surrounding liquid, even in presence of strongly colored drain fluids containing blood and/or bile (Figure 3d). In the same direction, by using pigments such as charcoal, TiO_2_ or BiVO_4_ and even organic dyes such as trypan blue (Figure 2h), the colors appear saturated and ensure a reliable naked eye readout even in heavily colored drain bag contents. However, contrary to structural colors, color from pigments does persist after digestion potentially causing coloration of the drain bag content (Figure 3d). Structural colorants offer additional design freedom, which in return can offer fluid relevant information (pH, salinity and more). Specifically, color changes of gold nanoparticle containing hydrogels depend on the distance between gold nanoparticles, something that can be controlled via the initial concentration of the gold nanoparticles, network architecture and the swelling characteristics.^[^ ^53^ ^]^

Changes of the sort can be a direct result of swelling originating from a measurable pH change (see Figure S8, Supporting Information, pH‐responsive colors). Robustness of the naked eye readout can be further engineered and optimized by tailoring the optical properties of the array's backing. For example, Figure 3e shows a sensor where the backing of the array provides additional discernibility features allowing to distinguish the state of the sensing elements using specific coloring or even the integration of fluorescent areas visible solely upon digestion of the corresponding sensing element. Altogether, this design freedom makes drain fluid monitoring highly observable and recognizable even in the case of complicated drain fluid matrices (e.g., due to the presence of blood and/or bile). Taken together, the capacity to continuously monitor and assess the severity of a leak (in conjunction with quantitative assays performed by hospital clinical chemistry) holds potential for alerting hospital staff about impending risks and guiding medical interventions during the rehabilitation period. As a result, a low‐barrier, non‐invasive tool, constantly in contact with drain effluents can equip medical staff with an additional patient surveillance tool, allowing close‐meshed and low‐cost patient monitoring.

### 2.3 Performance Evaluation Using Clinical Drain Fluid Samples

**Figure 4:**
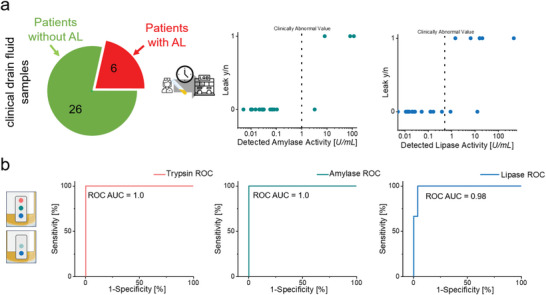
Overview of available clinical data and sensor ROC (N = 32 patient samples). a) Amylase and lipase activity of human samples as determined by clinical chemistry. In all anastomotic leak (AL) cases, elevated levels of enzyme activity were observed. b) Corresponding ROC curves for all major enzymes following exposure of the sensor to patient post‐operative effluents. The trypsin ROC was determined through contacting a trypsin responsive macromolecular sensor with human drain fluid for 24 h. The sensor correctly predicts a leak or its absence in 100% of the cases.

To evaluate the sensor's performance within a clinical context, we gathered 32 samples of drain effluents from patients having undergone abdominal surgery (for patient sample information, see Table S1, Supporting Information). These samples exhibited variations of volume, color, and consistency, reflecting the diversity inherent in clinical scenarios. Operating in a blinded study setting, the samples collected were analyzed both by the prepared macromolecular network sensor array (SensAL) and the clinical gold standard method (certified clinical chemistry lab determining the amylase and lipase activity levels). To assess the sensor response, the sensor was exposed to patient fluid for 24 h. Change in discoloration was recorded as a positive response while no visible change in sensing element consistency or color was indicative of a negative response. Among the 32 patients, six were clinically verified as suffering from a postoperative leak. Expectedly, the patients with a manifested leak demonstrated elevated amylase and lipase activity levels, following clinical chemistry evaluation, as can be seen in **Figure** **4**a. For all samples collected, the sensor array's response was in complete accordance with the clinical diagnosis, resulting in a 100% success rate. From the collected data, a Receiver Operating Curve (ROC) was calculated, resulting in a ROC with an AUC of 1.0 for trypsin and amylase, and an AUC of 0.98 for lipase (Figure 4b).

### 2.4 Continuous Leak Monitoring

**Figure 5:**
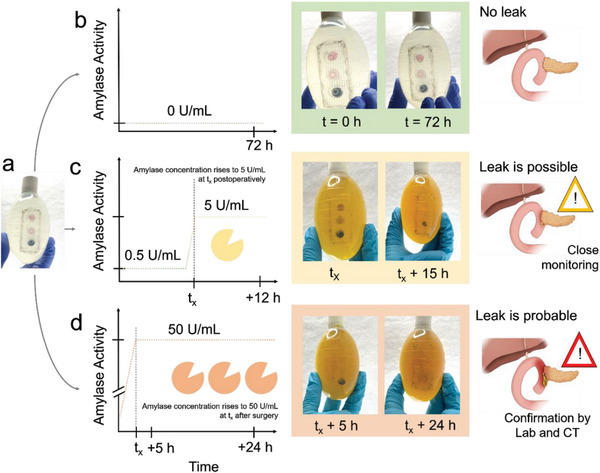
Anastomotic leak scenarios following pancreaticoduodenectomy. The sensor a) is exposed to fluids with changing enzyme activity. The scenario in (b) simulates a successful operation where no postsurgical AL occurred. The sensor remains intact even after 72 h. The scenario in (c) simulates a small leak occurring at time t_x_ after surgery. The sensor shows a clear change of the trypsin and amylase responsive sensing element t_x_ +15 h. The scenario in (d) simulates occurrence of a severe leak with high drain amylase levels at t_x_. In this case, a sensor response is visible within 5 h (t_x_ +5 h) for the trypsin and amylase sensor and a full sensor response after 24 h post leak (t_x_ +24 h). All leak simulation experiments performed at least three times using independent samples (N = 3).

To further demonstrate the usability of our macromolecular network sensor array in a simulated real‐world environment, we explored three different post‐operative leak scenarios that frequently occur after pancreaticoduodenectomy, a surgery notorious for its debilitating and life‐threatening postoperative complications.^[^ ^54^, ^55^, ^56^ ^]^ Current practices often entail the measurement of drain amylase levels on POD 1, 3, and sometimes POD 5.^[^ ^57^ ^]^ In stark contrast, the developed sensor array (**Figure** **5**a) facilitates a continuous monitoring approach. By enabling the ongoing surveillance of enzyme levels, as exemplified in our methodology, the timeline for detecting leaks can be minimized. This, in turn, allows for the timely initiation of leak interventions. This shift from intermittent measurements to a real‐time monitoring setup presents a decisive advancement.

In the first scenario (Figure 5b), the patient does not have a postsurgical AL. In this case, the sensor shows no response with all sensing elements being unreacted after 72 h of contact with the simulated drain fluid, demonstrating that the sensor array can be used for extended monitoring durations in human drain fluids. In the second scenario (Figure 5c), the mock patient showed initially a low baseline enzyme concentration (pancreatin, equivalent to 0.5 U mL^−1^ of amylase activity but also containing lipase and trypsin, Table S2, Supporting Information), which represents a typical level after surgery and flushing of the abdominal cavity. Literature shows that with these drain amylase levels, the risk of a leak or fistula is below 1%.^[^ ^58^ ^]^ Therefore this corresponds to a patient that does not suffer from a leak immediately after surgery. At t_x_ (for example, 12 h post‐surgery), however, a leak occurs and the corresponding drain amylase activity increases to 5 U mL^−1^ (high risk of anastomotic leak^[^ ^56^, ^59^ ^]^), co‐current with the occurrence of bile salts in the digestive effluent. Given the adapted conditions and despite a more pronounced drain fluid coloration, the sensor retains its naked eye readability, and all the sensing elements are easily discernable by the naked eye at t_x_. When exposing the sensor to these conditions at room temperature for an additional simulated hospital stay of 15 h, the amylase and the protease responsive sensing elements exhibit a complete reaction resulting in a distinct optical change, while the lipase responsive sensor network only partially responded.

We also conducted an experiment representing a severe leak immediately post‐surgery, shown in Figure 5d. Here, at the time of leak (t_x_) the amylase activity value rises to 50 U mL^−1^ aligning with levels faced a high probability of developing a postoperative pancreatic leak.^[^ ^60^ ^]^ While such high amylase levels likely coincide with other symptoms such as abdominal pain or fever, determining amylase levels with the current gold‐standard method may still take a significant amount of time due to the need of a sample being collected and analyzed by a centralized lab (if a sample is procured from the drain on POD 1 before the leak happens, it can take 48+ h until a leak is diagnosed (compare also Figure 1c)).^[^ ^61^ ^]^ In the case of the simulated severe leak, the sensor demonstrated a noticeable change indicative of a leak within 5 h after the leak (t_x_ + 5 h) at RT through the full reaction of the amylase and protease responsive sensing elements. This corresponds to a drastic reduction in detection time compared to current methods (5 h vs 12–48 h based on drain amylase in central labs on POD1 and POD3), and suggests feasibility of early detection of leaks at the bedside.^[^ ^57^ ^]^ After 24 h (t_x_ + 24 h) under this critical condition, the lipase responsive sensing element was also fully converted. Cementing the role of the lipase sensing elements as a dependable indicator for more severe leaks. This differentiation in digestion times combined with the above shown semi‐quantitative approach (see Figure 3) could significantly contribute to the differentiation of different leak severities. The outcomes of this experiment underscore the potential of the as designed electronic‐free sensor array, to effectively indicate even small leaks within the first few postoperative hours after their occurrence. While not every surgery on the GI tract involves the placement of a drain and placement of drains is frequently discussed,^[^ ^16^ ^]^ drains are routinely placed in patients undergoing pancreatic, esophageal and rectal surgeries in most centers in Europe and the US. The proposed sensor is applicable for patients with a drain, however, the integration of this sensors may alter the risk/benefit ratio of drains, something that requires assessment in upcoming investigations in the clinical setting.

## 3 Conclusion

In this work, we addressed one of the most dreaded complications in gastrointestinal surgery by designing a low‐cost (all materials can be acquired for low prices in bulk and require simple one step reactions to modify, resulting in materials costs of < 35 USD per kg (yielding ≈20.000 sensor elements), non‐electronic bed‐side patient monitoring approach fully compliant with today's clinical needs. We systematically designed and developed three distinct structurally colored enzyme‐responsive macromolecular network sensors, establishing a platform for straightforward, electronic‐free, continuous, and semi‐quantitative naked eye‐based detection of AL. The use of digestive enzymes as analytes and (structurally) colored macromolecular networks as recognition element and signal transducer enables both sensitive and specific detection of potential impending AL directly at the patient's bed‐side. Using clinical samples, the potential of the presented sensor technology is demonstrated in a highly relevant real world scenario. The sensor outperformed the prevailing clinical gold standard methods for post‐operative effluent assessment with regard to ease of detection and resource‐intensiveness, yielding equivalent diagnostic performance. Notably, the naked‐eye detection reduces the necessity for costly equipment or specialized personnel initially and can serve as an early warning of a potentially pre‐symptomatic leak, requiring follow‐up in‐depth investigations. It also attenuates the uncertainty and supports clinical decision making, regarding the need of further potentially costly diagnostic tests in central laboratories or imaging facilities (such as amylase detection in central clinical chemistry labs, and/or confirmatory CT). Nonetheless, it is important to acknowledge that further investigations, encompassing a broader patient and leak cohort, are imperative to affirm the sensor's accuracy and clinical utility.

On a larger scale, this low‐cost sensing approach offers a promising way to efficiently identify patients at risk in a resource‐efficient manner. This is especially relevant in an outpatient setting, where patients are at risk for late‐onset leaks.^[^ ^14^ ^]^ It guides physicians by providing confidence in performing additional established, more costly diagnostic tests (such as fluoroscopy, CT, MRI, or others), or in deciding on nutritional protocols (i.e., the early re‐initiation of enteral nutrition after surgery). Due to the straightforward interpretability of the sensor read‐out and the minimal human intervention needed, this approach seamlessly integrates into hospital routines with minimal resource consumption. Consequently, the sensor array presented herein has the potential to play a pivotal role in effectively tackling the challenges of both over‐ and under‐diagnosis of leaks, as well as resource management in today's healthcare system. This new detection method, therefore, holds the potential to significantly enhance patient outcomes while contributing to economic efficiency.

## 4 Experimental Section

### Materials

All materials were purchased from Sigma–Aldrich (Merck). Acrylamide (AAm) monomers and PEG‐DA were used without further purification. Human samples were received from the University Hospital Zurich (USZ, approved by the Cantonal Ethics Commission of the Canton of Zurich, Switzerland, BASEC no. Req‐2022‐01026).

### Gelatin‐MA Synthesis and Hydrogel Formation

For the synthesis of Gelatin‐MA a slightly modified version of the procedure from Dubruel et al.^[^ ^42^ ^]^ was used (see Scheme S1, Supporting Information). In brief, to a 100 mL two‐necked round bottom flask equipped with a reflux condenser, stir bar, and a stopcock, 50 mL of PBS (Dulbecco modified without CaCl_2_ and MgCl_2_) were added. While stirring vigorously, 5 g of gelatin (type A from porcine skin) were added and the mixture was heated to 40 °C (oil bath) and stirred until the gelatin was fully dissolved (15–20 min). Afterward, 580 µl of methacrylic anhydride was added via syringe and stirring was continued for 3 h. During this period the color changed to light brown. Next, 50 mL Milli‐Q water (MQ, Metrohm) was added and stirring was continued for an additional 20 min. The whole mixture was then pipetted into dialysis tubing (3.5 kDa cutoff Servapor) and dialyzed against deionized water for 2 days at RT with frequent water changes. Afterward the solution was frozen, freeze‐dried and a white crystalline foam was obtained.

For the preparation of various Gel‐MA hydrogels used in digestion experiments, 5 wt.% gelatin was dissolved in either water or gold nanoparticle suspension (prepared as reported by Puntes et al. with one growth step and two‐time centrifuging at 15.000 × g for 10 min and consecutive removal of half of the supernatant to concentrate the suspension^[^ ^62^ ^]^) by stirring and heating on a hotplate set to 50 °C until everything was dissolved. Afterward, 10 vol% Lithium phenyl‐2,4,6‐trimethylbenzoylphosphinate (LAP, 6.33 mg mL^−1^ in MQ) was added and briefly stirred. The mixture was then either pipetted into Teflon molds (500 µl) or evenly spread on a glass plate to form a thin film of ≈1 mm (see Figure S6, Supporting Information). The whole glass plate was then placed in a fridge for 20 min and the plate was irradiated with UV light (UVASPOT 400/T mercury lamp, Hönle) for 5 min at a distance of 15 cm. Next, a 9 mm biopsy punch was used to stencil out circular elements, which were then kept in the fridge for several days until further use.

### Starch Acrylate Synthesis and Hydrogel Formation

For this synthesis, a 4‐(Dimethylamino)pyridine (DMAP) catalyzed synthesis was performed, similar to the method used by Kessler et al.^[^ ^41^ ^]^ to functionalize alcohols (see Scheme S2, Supporting Information). To a 250 mL three‐necked round bottom flask, equipped with a stir bar, reflux condenser, and a thermometer, 4 g of starch (from potatoes, Sigma Aldrich) as well as 150 mL of Milli‐Q was added. After stirring briefly, 27 mg of DMAP was added and the mixture was heated to 50 °C (inside the flask) for 15 min to achieve partial swelling of the starch granules. Next, the mixture was cooled to 40 °C and 1.25 mL methacrylic anhydride were added dropwise. During the whole reaction, the pH of the solution was carefully monitored. After 2 h, the pH of the reaction had dropped to 4–5. By adding 5 m NaOH, the pH was adjusted to 6. One hour later the pH was readjusted by addition of more 5 m NaOH upon which the reaction pH was ≈9. The reaction mixture was then stirred overnight in the dark. The next day, the solution was pipetted into dialysis tubing (3.5 kDa cutoff, Servapor) and dialyzed against 10 L of deionized water. The water was changed daily for 3 days. After freezing the solution, it was freeze dried and a white powder was obtained. To produce the starch hydrogels, 5 wt.% starch was suspended gold nanoparticle suspension (see Gel‐MA), together with 15 wt.% acrylamide. After short vortexing, the mixture was transferred to an oven and kept at 80 °C until it turned into a viscous liquid. Meanwhile a glass plate was preheated to 50 °C. Next, LAP (6.33 mg mL^−1^) was added to the mixture and briefly vortexed. The mixture was then transferred on a warm glass plate and a thin film was made followed by immediate polymerization by UV light for 5 min (UVASPOT 400/T mercury lamp, Hönle). Then, a 9 mm biopsy punch was used to stencil out circular elements, which were then kept in the fridge for several days until further use.

### Plant‐Oil Oleogel Synthesis

Plant‐oil based oleogels were synthesized using procedures inspired by Marangoni et. al.^[^ ^39^ ^]^ as well as Mohammadifar et al.^[^ ^49^, ^63^ ^]^ Coconut oil with 5 wt.% of ethylcellulose (300 cp) and optionally 2 wt.% active charcoal was added to a 50 mL Erlenmeyer flask. The mixture was stirred in an oil bath at 180 °C for 2 h. The mixture was then poured on a glass plate to form a thin film (≈1 mm in thickness) and the glass plate was transferred to a fridge to cool. Once the film solidified, a 9 mm biopsy punch was used to stencil out circular elements, which were then kept in the fridge for several days until further use.

### Digestion of Gels, Time Series, and Concentrations

For all digestion experiments a similar setup was used. Circular elements were put into 12‐well plates (Starch‐MA and Gel‐MA, 3 mL total end‐volume) or into 6‐well plates (Oleogel, 6 mL total end‐volume) and the corresponding amount of enzyme (amylase Type VI‐B ≥ 5 units/mg, lipase Type II ≥ 125 units/mg and trypsin from porcine pancreas) was added via a 1 mg mL^−1^ stock solution or directly as dry powder until the right concentration in the well was achieved. The plates were then put onto an orbital shaker and either placed into an oven (37 °C, body temperature) or kept at RT. To collect images, a tablet (ipad) and an app (Skyflow – Time‐lapse shooting) was used to take pictures every 10 min from a 20–40 cm distance. Digestion was then determined through manual image analysis by a blinded operator. The sensing elements have excellent batch to batch repeatability. For the kinetic characterization data as well as for the test with the clinical samples, over 5 different batches produced over 6 months were used. Additionally in the kinetics experiments technical triplicates from the same batch as well as replicates from different batches were compared. All materials showed the same response and no statistically significant differences were observed.

### Clinical Samples

Samples were collected from University Hospital Zurich (USZ) with an ethics approval in place (BASEC no. Req‐2022‐01026). Volumes, colors as well as consistencies of the samples varied. Type of operation/complication, date collected and postoperative day the sample was noted in anonymized form and the study was performed in a blinded setting (clinician holding the key). Samples were split into two parts. From each sample, 3–10 mL were decanted into heparin coated tubes that were then frozen until analysis by the Institute for Clinical Chemistry (IKC) of USZ, where amylase and lipase activity were determined. Furthermore bilirubin content was measured. The rest of the sample (varying volume) was then added to a falcon tube into which a 500 µL Gel‐MA sensor (1% PEG‐DA‐AuNP) was added. The samples were then incubated overnight at 37 °C while shaking gently on an orbital shaker. Digestion/reaction was determined by a blinded operator the next day and again after 24 h in the case of unreacted sensing elements.

### Prototype Proof of Principle Experiments

Prototype gel holders were first 3D printed and then used as negative molds for hot pressing plastic sheets over them. The molds were open on one side and had a hole over each of the sensing elements on the other side. Sensing elements were then transferred into the plastic forms and a net was attached to the backside in order to avoid the sensing elements slipping out of the mold. The net also served as an attachment point for additional backings. For the digestion experiment a Jackson Drain (provided by USZ) was used. The cap was removed in order to place the macromolecular network sensor strip inside. The drain bag was then filled with 100 mL PBS (Dulbecco modified with CaCl_2_ and MgCl_2_) and enzymes in the corresponding concentration were added. A septum was hot‐glued on top of the drain to seal it and the bag was gently mixed with an orbital shaker for the above described time periods. For the 5 and 50 U mL^−1^ experiments, a bile (bile extract porcine) concentration of 5.3 mg mL^−1^ was used.

### Statistical Analysis

No data preprocessing was applied. All experiments were performed at least three times using independent samples, unless otherwise stated. Experiments were performed in blinded settings whenever applicable. Data is represented as mean ± standard deviation, unless otherwise stated. ROC values were calculated with Origin. Statistical analysis was performed with Origin and Excel.

## Conflict of Interest

Alexander Jessernig, Alexandre H.C. Anthis, and Inge K. Herrmann declare inventorship on a patent filed by ETH Zurich and Empa (EP23203384). All other authors declare no conflict of interest.

## Author Contributions

I.K.H. conceived the project idea. A.J. developed the macromolecular sensing elements, performed all experimental investigations, and prepared the first draft of the manuscript with support from A.H.C.A. and I.K.H. A.H.C.A. contributed to the sensing element designs and device integration. E.V. helped with experiments. J.W. provided clinical samples. J.W., J.R., V.L., and A.A.S contributed with their clinical expertise. All authors contributed to discussion, data interpretation and manuscript writing.

## Supporting information

Supporting Information

## Data Availability

The data that support the findings of this study are available from the corresponding author upon reasonable request.
