# Supplementary material for: Early Detection and Monitoring of Anastomotic Leaks via Naked Eye‐Readable, Non‐Electronic Macromolecular Network Sensors
Source: Adv Sci (Weinh). 2024 May 22;11(29):2400673. doi: 10.1002/advs.202400673 (PMC11304232; doi:10.1002/advs.202400673)
Supplement: Supplementary file 1 — Supporting Information [file ADVS-11-2400673-s001.pdf]

ADVANCED SCIENCE
Open Access

Supporting Information

for *Adv. Sci.*, DOI 10.1002/advs.202400673

Early Detection and Monitoring of Anastomotic Leaks via Naked Eye-Readable, Non-Electronic Macromolecular Network Sensors

*Alexander Jessernig, Alexandre H.C. Anthis, Emilie Vonna, Jachym Rosendorf, Vaclav Liska, Jeannette Widmer, Andrea A. Schlegel and Inge K. Herrmann**

SUPPORTING INFORMATION

Early Detection and Monitoring of Anastomotic Leaks via Naked Eye-readable, Non-electronic Macromolecular Network Sensors

Alexander Jessernig,[1,2] *Alexandre H.C. Anthis,* [1,2] *Emilie Vonna,* [1] *Jachym Rosendorf,*[4,5] *Vaclav Liska,* [4,5] *Jeannette Widmer,*[3] *Andrea A. Schlegel,*[6] *Inge K. Herrmann*[1,2,7,8]*

[1] Nanoparticle Systems Engineering Laboratory, Institute of Energy and Process Engineering (IEPE), Department of Mechanical and Process Engineering (D-MAVT), ETH Zurich, Sonneggstrasse 3, 8092 Zurich, Switzerland.
[2] Particles-Biology Interactions Laboratory, Department of Materials Meet Life, Swiss Federal Laboratories for Materials Science and Technology (Empa), Lerchenfeldstrasse 5, 9014 St. Gallen, Switzerland.
[3] Department of Surgery and Transplantation, Swiss HPB Centre, University Hospital Zurich, 8091 Zürich, Switzerland.
[4] Department of Surgery, Faculty of Medicine in Pilsen, Charles University, Alej Svobody 923/80, Pilsen, 32300 Czech Republic.
[5] Biomedical Center, Faculty of Medicine in Pilsen, Charles University, Alej Svobody 1655/76, Pilsen, 32300 Czech Republic.
[6] Transplantation Center, Digestive Disease and Surgery Institute and Department of Immunity and Inflammation, Lerner Research Institute, Cleveland Clinic, 9620 Carnegie Ave, Cleveland, OH, 44106 United States.
[7] The Ingenuity Lab, University Hospital Balgrist, Balgrist Campus, Forchstrasse 340, 8008, Zurich, Switzerland.
[8] Faculty of Medicine, University of Zurich, Rämistrasse 74, 8006 Zürich, Switzerland.

*inge.herrmann@empa.ch; ingeh@ethz.ch; ; inge.herrmann@uzh.ch

+41 (0)58 765 7153

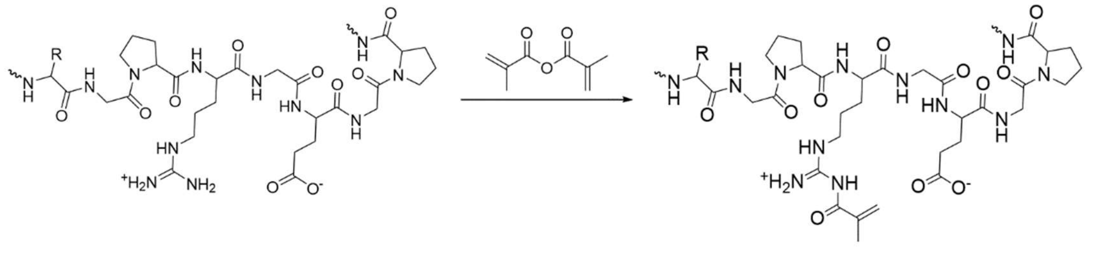

Figure S1: Reaction scheme of the methacrylation of gelatin. Using methacrylic anhydride, amine and guanidine containing side chains of amino acids are methacrylated, resulting in a photocrosslinkable gelatin.

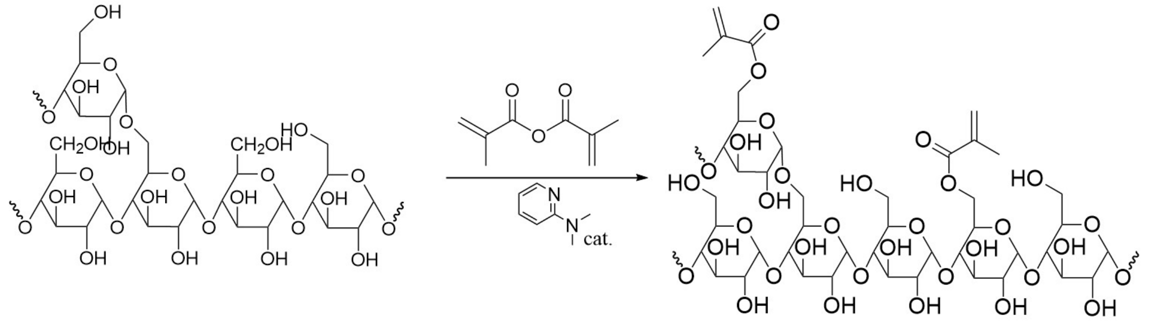

Figure S2: Reaction scheme of the methacrylation of starch. Using methacrylic anhydride, alcohol groups on starch are methacrylated, resulting in a photocrosslinkable starch.

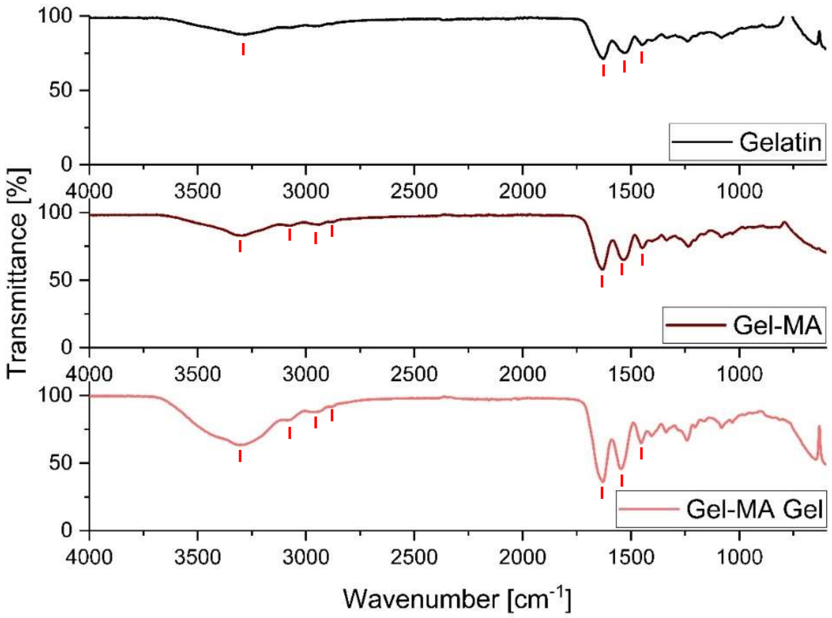

Figure S3: IR-spectra of gelatin, Gel-MA and its corresponding hydrogel. The spectrum of gelatin is similar to literature with the major peaks corresponding to the various amide bonds found in this biomolecule.[56] While in the spectra of Gel-MA and the Gel-MA Gel, the methacrylate C=C bands are overlapped by the characteristic amide bond bands, the spectra are in accordance with literature.[57,58]

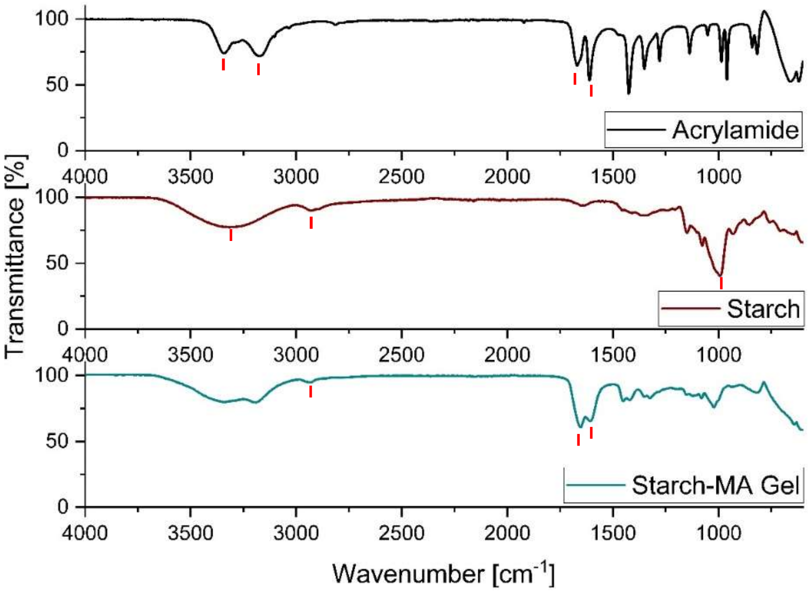

Figure S4: IR spectra of Acrylamide, Starch and the Starch-MA hydrogel. The acrylamide spectra is in accordance with literatures. The two bands from 3200-3400 cm^{-1} correspond to the primary amine found in acrylamide. While the bands around 1650 cm^{-1} correspond to the amide C=O stretch vibration and the band around 1600 cm^{-1} corresponds to the $NH_2\delta$ vibration.[57,59] *The spectrum of starch is good accordance with literature. The strong bands around 1000 cm^{-1} correspond to the various C-O and C-C stretch modes while the broad band from 3000-3600 cm^{-1} stems potentially from adsorbed water as well as the O-H stretch vibration of the alcohol groups found in starch. The band around 2900 cm^{-1} likely corresponds to the C-H stretch modes.*[57,60] *The Starch-MA hydrogel spectrum shows a good superposition of the two materials.*

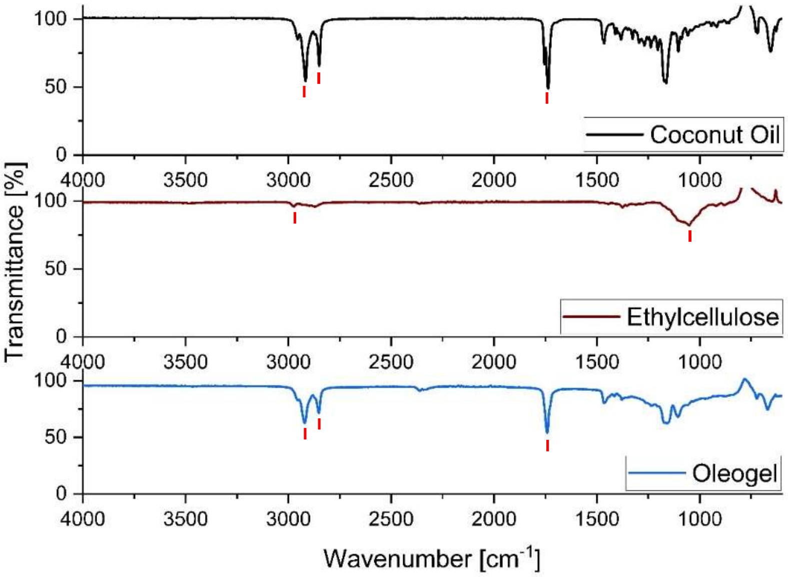

Figure S5: IR spectra of coconut oil, ethylcellulose and the resulting oleogel. The coconut oil displays two strong bands around 2900 cm^{-1} corresponding to the C-H stretch vibrations of the alkane chains and another strong band around 1750 cm^{-1} corresponding to the C=O stretch mode of the ester bonds found in the tryglyceride.[57] *While the bands found in the ethylcellulose spectrum are weak, is shows similarity to the starch spectrum. The bands around 1000 cm^{-1} correspond to the various C-O and C-C stretch modes and the band around 2950 cm^{-1} stemms from the C-H stretch modes.*[57] *The spectrum of the oleogel has more similarity to the spectrum of coconut oil and the gel only contains 5 wt% ethylcellulose. However, the oleogel spectrum still shows a band broadening around 1000 cm^{-1} stemming from the ethylcelulose.*

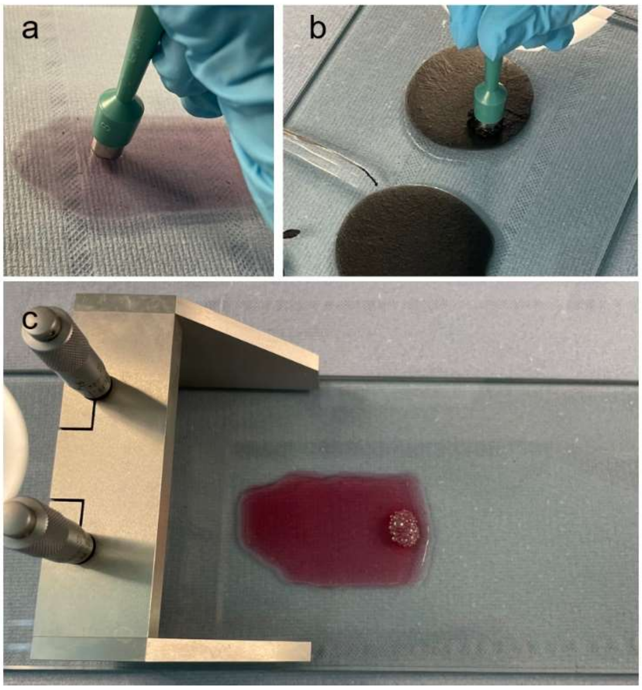

Figure S7: Thin film fabrication and macromolecular network sensing elements manufacturing of Starch-MA (a), Oleogel (b) and Gel-MA (c). A biopsy punch was employed to excise sensing elements.

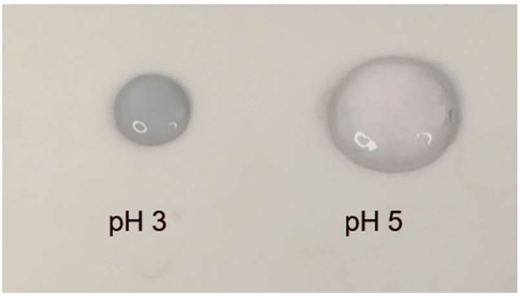

Figure S8: Swelling behavior of 25% acrylic acid hydrogels after immersion in different buffer solutions. Due to bigger distances between the gold particles a color change is visible.

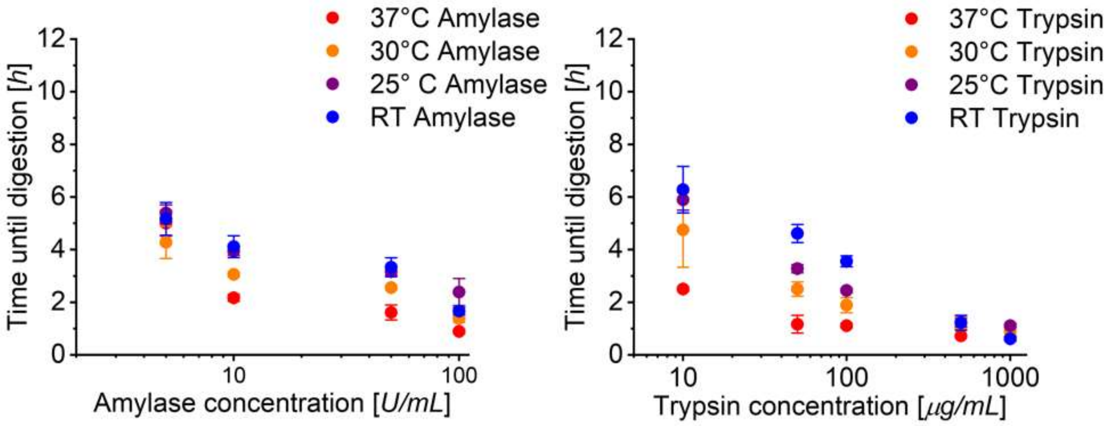

Figure S9: Additional temperature data of Gel-MA and Starch-MA macromolecular sensing elements.

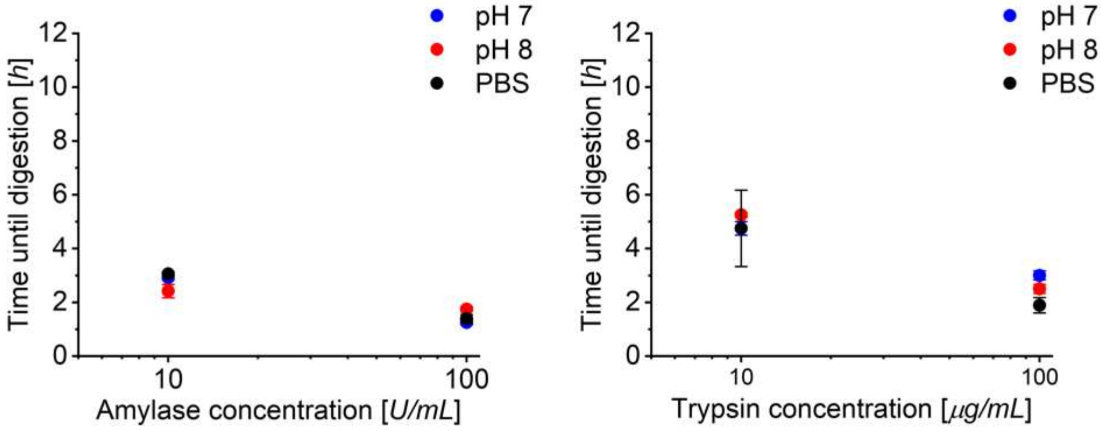

Figure S10: pH dependence of Gel-MA and Starch-MA macromolecular sensing elements at 30°C.

Table S1: Enzyme activity of patient samples obtained from the University Hospital Zurich. From each patient the amylase and lipase activity was determined and it was noted whether or not a leak occurred. Patient #1 underwent total pancreaticoduodenectomy, the removal of the pancreas, during which several complications occurred, and a drain was placed directly into the GI tract of said patient. Therefore, the case does not classify as a leak. The heightened amylase and lipase levels are explained by the fact that through this procedure the patient is reliant on an external source of enzymes since his body can no longer produce them. The missing trypsin sensor response is explained by the fact, that the sensor used detects trypsin which the patient cannot produce themselves anymore and did not get supplemented with at the time.

PATIENT #	TYPE OF SURGERY	AMYLASE [U/L]	LIPASE [U/L]	LEAK? (Y/N)
**1**	Pancreatectomy	3264	12604	n
**2**	Duodenal perforation	<10 icteric	15819	y
**3**	Abdominal cavity reconstruction	too viscous	128	n
**4**	Biliary stoma	<10 icteric	6462	y
**5**	Sigma resection	27	15	n
**6**	Splenectomy (potentially damage to pancreas tip)	22	12	n
**7**	Abdominal cavity reconstruction	11	13	n
**8**	Stomach perforation	15	14	n
**9**	multiple small intestine perforations, pancreas fistula	115640	455300	y
**10**	Ileo-trasverse anastomosis	32	52	n
**11**	Rectosigmoid resection and primary descendorectostomy	29	11	n
**12**	Gastric resection+ reconstruction	82	383	n
**13**	Small intestine perforation	21	52	n
**14**	Whipple procedure	<10	5	n
**15**	ALPPS (Associating Liver Partition and portal vein ligation for staged hepatectomy)	not possible	15694	y
**16**	Stomach perforation	57	27	n
**17**	Whipple procedure	too viscous	too viscous	n
**18**	Whipple procedure	<10	21	n
**19**	Hepaticojejunostomy	105	893	n

**20**	Frey Procedure	30	13	n
**21**	Frey Procedure	28	15	n
**22**	Abscess after echinococcus cyst	8125	1436	y
**23**	Liver Transplant	<10	21	n
**24**	Whipple revision	82201	20580	y
**25**	Whipple procedure	66	123	n
**26**	ALPPS (Associating liver partition and portal vein ligation)	31	20	n
**27**	Sigma resection, rectosigmoidectomy, livermetastasis resection	<10	24	n
**28**	Tumor resection of pancreas (anterior), duodenum and liversegment resection	10	15	n
**29**	Small intestine segmental resection	<10	11	n
**30**	Hemikoelctomy	<10	15	n
**31**	Hemikoelctomy	5	13	n
**32**	Pancreas Transplant	70	164	n

Table S2: Enzyme activity of pancreatin used for leak simulation experiments as determined by clinical chemistry.

**Enzyme**	**Activity**
Amylase	9.76 ± 0.97 U/mg
Lipase	1.67 ± 0.20 U/mg
